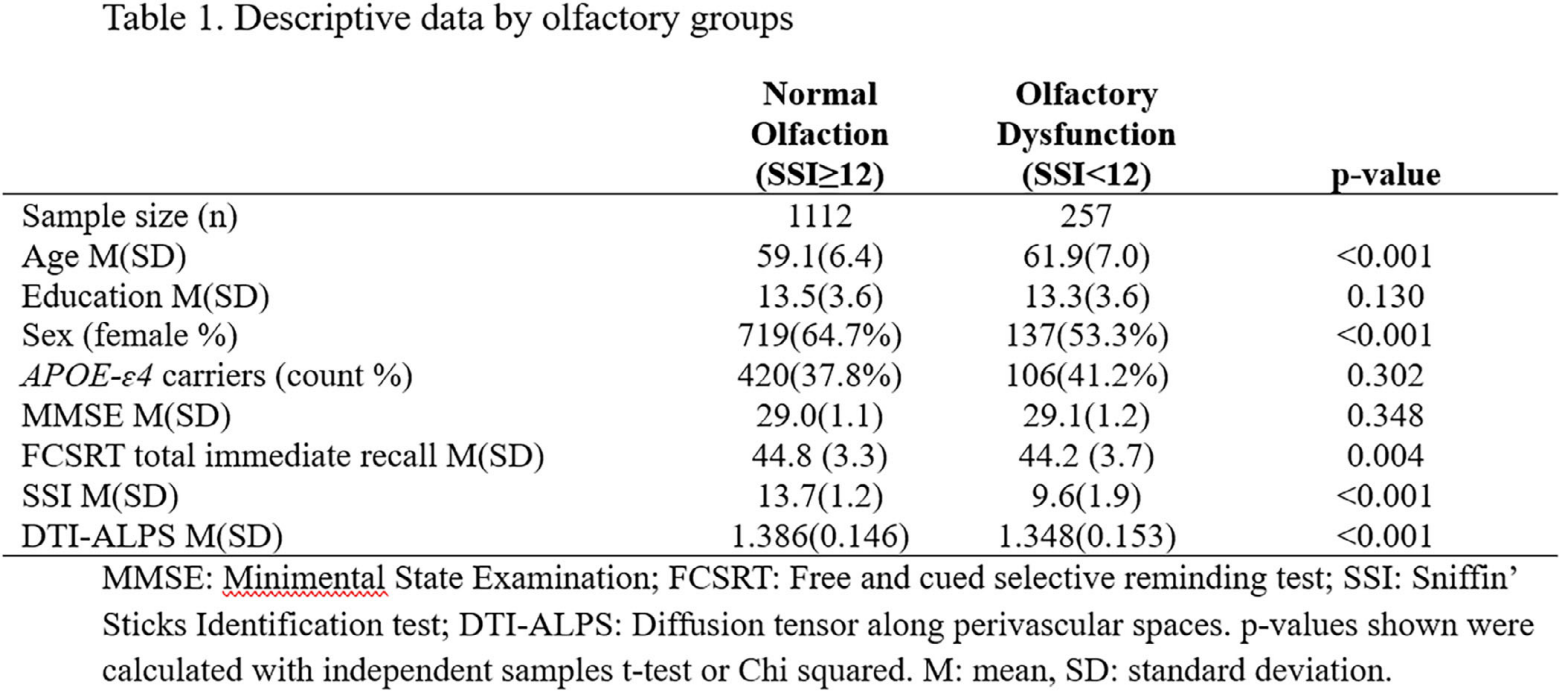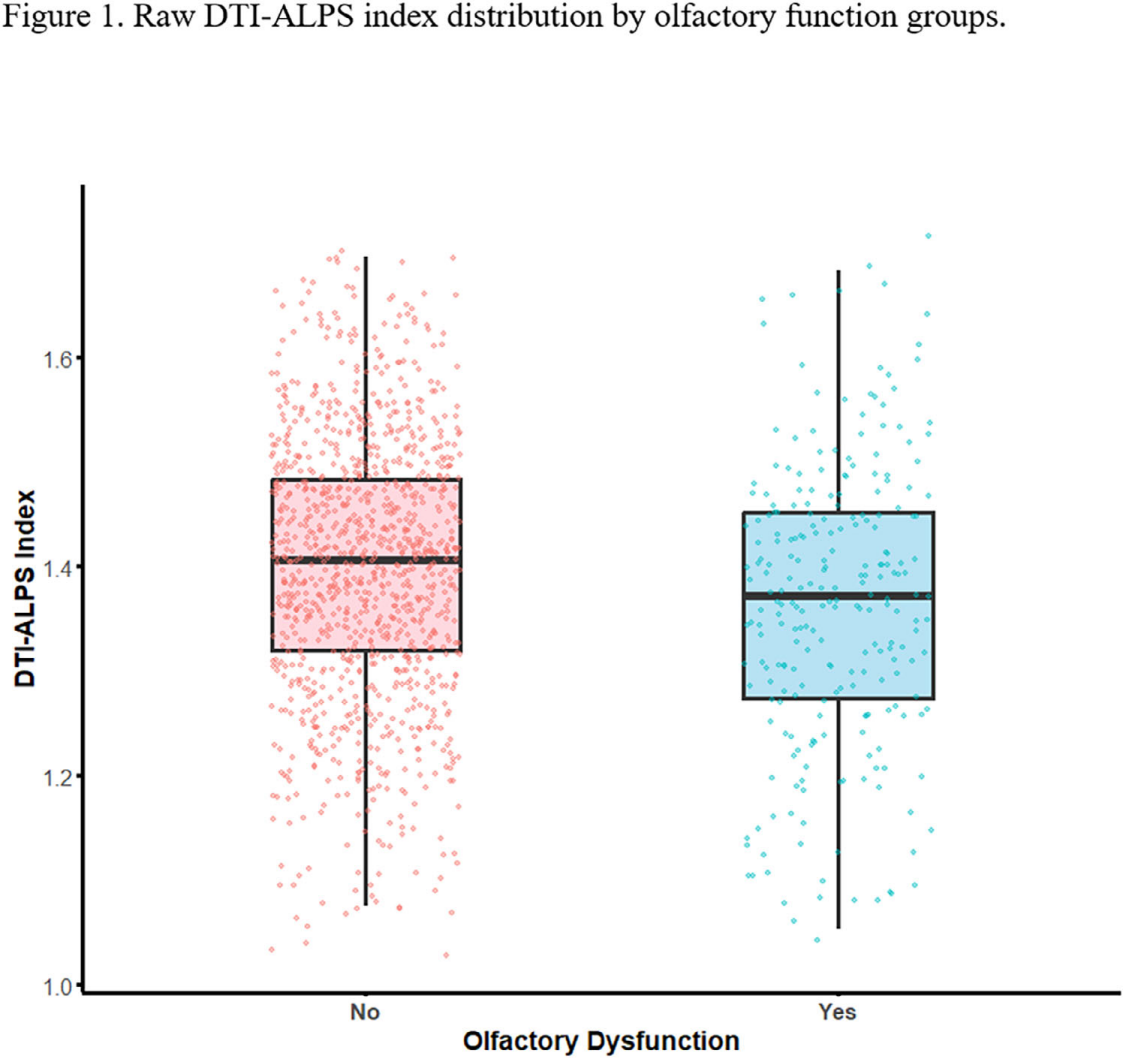# Olfactory dysfunction relates to lower glymphatic functioning as measured by DTI‐ALPS in cognitively unimpaired individuals with family history of dementia

**DOI:** 10.1002/alz70857_102197

**Published:** 2025-12-25

**Authors:** Gonzalo Sánchez‐Benavides, Mariateresa Buongiorno, Michalis Kassinopoulos, Anna Brugulat‐Serrat, David López‐Martos, David Vállez García, Natalia Vilor‐Tejedor, Juan Domingo Gispert, Gemma Salvadó, Oriol Grau‐Rivera

**Affiliations:** ^1^ Barcelonaβeta Brain Research Center (BBRC), Pasqual Maragall Foundation, Barcelona, Spain; ^2^ Centro de Investigación Biomédica en Red de Fragilidad y Envejecimiento Saludable (CIBERFES), Instituto de Salud Carlos III, Madrid, Spain; ^3^ Hospital del Mar Research Institute (IMIM), Barcelona, Spain; ^4^ Hospital Universitari Vall d'Hebron, Barcelona, Spain; ^5^ Hospital del Mar Research Institute, Barcelona, Spain; ^6^ Barcelonaβeta Brain Research Center, Barcelona, Spain; ^7^ Department of Genetics, Radboud Medical University Center, Nijmegen, Netherlands; ^8^ Centre for Genomic Regulation (CRG), Barcelona Institute of Science and Technology (BIST), Barcelona, Spain; ^9^ AstraZeneca, Barcelona, Spain; ^10^ Clinical Memory Research Unit, Department of Clinical Sciences Malmö, Lund University, Lund, Sweden; ^11^ Department of Clinical Sciences, Clinical Memory Research Unit, Lund University, Lund, Spain; ^12^ Centro de Investigación Biomédica en Red de Fragilidad y Envejecimiento Saludable (CIBERFES), Instituto de Salud Carlos III, Barcelona, Spain; ^13^ Servei de Neurologia, Hospital del Mar, Barcelona, Spain

## Abstract

**Background:**

Olfactory dysfunction (OD) is common in elders and almost ubiquitous in patients with neurodegenerative diseases. Early accumulation of pathological protein aggregates in the olfactory bulb and related structures may relate to impaired solute clearance by the glymphatic system ‐ brain waste clearance system mainly active during sleep‐. We aimed to explore the association between olfactory performance and diffusion tensor imaging along perivascular spaces (DTI‐ALPS) as a proxy of glymphatic function in cognitively unimpaired (CU) individuals with family history of dementia.

**Method:**

We analyzed data from 1,369 ALFA cohort participants (age range 47‐77 yo, mean[SD]:59.6[6.5], 62.5% women) with family history of dementia (88.5% Alzheimer's disease). Olfaction was assessed with the 16 item‐Sniffin’ Sticks identification (SSI) test. DTI‐ALPS index was computed from diffusion‐weighted MR images. OD was defined using a standard cut‐off (SSI scores <12,). We explored the association between DTI‐ALPS and presence of OD using logistic regression, with OD as dependent variable, high/low median split DTI‐ALPS groups as predictor, and age, education, sex, and *APOE*‐ɛ4 status as covariates. Due to the ceiling effect shown by SSI scores, a Generalized Linear Model (GLM) with a log‐link function was used to explore the associations between continuous SSI scores and DTI‐ALPS measures adjusted by covariates.

**Result:**

OD was present in 18.8% of participants and associated with older age (OR: 1.06 [1.04–1.09]; *p* < 0.001), male sex (OR: 1.51 [1.14–2.01]; *p* =  0.004), and lower DTI‐ALPS scores (OR: 1.35 [1.01–1.80]; *p* =  0.04). In stratified analyses, similar albeit non‐significant trends were observed for both sexes (female OR:1.37 [0.94‐2.01]; *p* = 0.1; male OR:1.34 [0.86‐2.11]; *p* = 0.2). DTI‐ALPS scores were positively associated with continuous SSI scores (est=1.084; *p* = 0.007), particularly in individuals without OD (est=1.04; *p* = 0.02).

**Conclusion:**

CU individuals with low performance on a short olfactory identification test exhibit reduced glymphatic function as measured by DTI‐ALPS. This association was observed even in the normal olfactory range. Further longitudinal studies are needed to validate OD as a potential clinical marker of glymphatic dysfunction in individuals at risk of cognitive impairment.